# Heatwaves and Hospital Admissions for Mental Disorders in Northern Vietnam

**DOI:** 10.1371/journal.pone.0155609

**Published:** 2016-05-19

**Authors:** Phan Minh Trang, Joacim Rocklöv, Kim Bao Giang, Gunnar Kullgren, Maria Nilsson

**Affiliations:** 1 Department of Public Health and Clinical Medicine, Unit of Epidemiology and Global Health, Umeå University, Sweden; 2 Institute for Preventive Medicine and Public Health, Hanoi Medical University, Vietnam; 3 Department of Psychiatry Clinical Sciences, Umeå University, Umeå, Sweden; Public Health Agency of Canada, CANADA

## Abstract

Studies in high-income countries have shown an association between heatwaves and hospital admissions for mental disorders. It is unknown whether such associations exist in subtropical nations like Vietnam. The study aim was to investigate whether hospital admissions for mental disorders may be triggered, or exacerbated, by heat exposure and heatwaves, in a low- and middle-income country, Vietnam. For this, we used data from the Hanoi Mental Hospital over five years (2008–2012) to estimate the effect of heatwaves on admissions for mental disorders. A zero-inflated negative binomial regression model accounting for seasonality, time trend, days of week, and mean humidity was used to analyse the relationship. Heatwave events were mainly studied as periods of three or seven consecutive days above the threshold of 35°C daily maximum temperature (90^th^ percentile). The study result showed heatwaves increased the risk for admission in the whole group of mental disorders (F00-79) for more persistent heatwaves of at least 3 days when compared with non-heatwave periods. The relative risks were estimated at 1.04 (0.95–1.13), 1.15 (1.005–1.31), and 1.36 (1–1.90) for a one-, three- and seven-day heatwave, respectively. Admissions for mental disorders increased among men, residents in rural communities, and the elderly population during heatwaves. The groups of organic mental disorders, including symptomatic illnesses (F0-9) and mental retardation (F70-79), had increased admissions during heatwaves. The findings are novel in their focus on heatwave impact on mental diseases in a population habituating in a subtropical low- and middle-income country characterized by rapid epidemiological transitions and environmental changes.

## Introduction

In recent years, climate change has become a global challenge with its impacts on human health due to the exposure to weather variations and extreme weather, such as heatwaves, floods, storm-surges, droughts, with increased morbidity as well as mortality worldwide [1–3].

High temperature can cause morbidity and mortality and people at high risk include: the elderly, children, and persons with pre-existing chronic diseases, such as cardiovascular disease and respiratory or mental disorders [4]. Psychological and mental health implications of heatwaves in relation to climate change have in recent years been researched by the health science community [4–7]. Aspects of human life involving physical and mental health, including trauma, chronic stress, anxiety, depressive disorder and comorbidity of psychopathology or medical illness, can be directly and/or indirectly impacted by the consequences of heatwaves [5, 7]. Higher risks of delirium and neuropsychiatric syndromes, with symptoms comprising altered consciousness, agitation, restlessness, unconsciousness, and even death among populations suffering from heatstroke due to heatwaves, have been shown [5]. Some epidemiological and experimental studies showed a relationship between heat and violence, increasing rates of assault, rape and robbery owing to rising global temperatures [8, 9]. This may be related to more stress hormones released into the blood, a result of excessive exposure to heat [10]. In addition, an increase in alcohol consumption, drug abuse, violence, family break-ups and suicide has been reported during periods of heatwaves as well as after extreme weather events, particularly in rural and semi-rural areas [7, 11, 12].

Most research on the effects of high temperature on psychiatric disorders has been conducted in developed nations [6,7,13–16]. Several reports from Australia argued that extreme heat events in 2007–2009 had direct and delayed impacts on mental health and social issues, increasing the prevalence of depression, anxiety and substance abuse [6, 7, 11, 12, 17, 18]. Studies in Adelaide, Australia, showed a positive relationship between ambient temperature and hospital admissions for mental and behavioural disorders, with higher rates of dementia, mood (affective) disorders, neurotic, stress and somatoform disorders, disorders of psychological development, and senility, especially in the age group of 65–74 years during heatwaves [7, 19, 20].

Several studies from Scotland, Ireland, Canada and Israel reported that mean maximal monthly environmental temperature impacted on psychotic exacerbation in patients with schizophrenia [16, 21–25]. Moreover, there were correlations between seasonality, mean daily temperature range, ward temperature and incidence or severity of symptoms in admissions for schizophrenia [23, 26].

During the last decade, scientists have considered the influence of temperature in the physiopathology of various neurodegenerative and psychiatric disorders. Several studies have proven an association between brain temperature and mood disorders–for example, that the brain temperature may rise with mania and fall with depression [27]. Some studies showed that the peak of admissions for mania was in the summer season with high temperatures, while the number of cases for depression increased in the winter season [28–30]. Failure in temperature control mechanisms has been shown to be related to mental disorders. For example, patients with schizophrenia exhibit dysregulation of the body temperature [26, 31]. Moreover, neurotransmitters, such as dopamine and serotonin, have shown an association with the central control of thermoregulation in humans [32–38].

So far, few studies have assessed the relationship between heatwaves, and the length of heat episodes, with hospital admissions for mental disorders, in all-causes and cause-specific groups of mental illness [25]. Vietnam is undergoing rapid changes, manifested by urbanization, epidemiological and demographic transitions, and health system development [39, 40] and it is important to understand how the transitions interact with environment and diseases. Studies are needed that identify the relationship between heat exposure and hospital admissions for mental disorders, and that identify groups susceptible to mental illness from heat exposure. Such research is necessary to improve public health during development and climate change, and may have bearings for similar countries facing epidemiological transitions and environmental changes.

## The Study Aim

The study aim was to investigate whether hospital admissions for mental disorders may be triggered, or exacerbated, by heat exposure and heatwaves, in Hanoi, Vietnam.

## Method and Materials

### Hospitalization data

Hanoi Mental Hospital is one of two big mental hospitals taking care of mental patients in Hanoi City. Both inpatients as emergency visits and outpatients in Hanoi and neighbouring provinces have access to it. In this study, we considered a database from Hanoi Mental Hospital covering mainly emergency cases for mental disorders in Hanoi City.

A database from Hanoi Mental Hospital covering 5 years from 2008 to 2012 was used including all patients with mental health disorders in the north of Vietnam (including Hanoi). The hospital admissions were diagnosed according to the International Classification of Diseases 10 (ICD 10) by mental health specialists from the hospital. Mental health disorders were classified according to the ICD 10 in groups of the diagnosis codes: F00 to F99. In this study we divided the group of mental disorder (F) into eight groups:

1. Organic mental disorders, including symptomatic illnesses (F00-F09),
2. Mental and behavioural disorders due to psychoactive substance use (F10-19),
3. Schizophrenia, schizotypal, and delusional disorders (F20-29),
4. Mood (affective) disorders (F30-39),
5. Neurotic, stress-related, and somatoform disorders (F40-48),
6. Behavioural syndromes associated with physiological disturbances and physical factors (F50-59),
7. Disorders of adult personality and behaviour (F60-F69),
8. Mental retardation (F70-79).

Admissions for mental disorders in Hanoi Mental Hospital during five years (2008–2012) included all diagnostic groups, except F60-F69. Thus, in this study, we researched hospital admission in relation to heatwaves for seven groups of mental disorders reported by the hospital.

### Climate and meteorological data

Located at 21°2′0″N 105°51′00″E, Hanoi has typically subtropical weather with four defined seasons. Hanoi’s area of 3.324,92 km^2^ includes 13 districts (referred to as the urban area) and 17 suburbs (referred to as the rural area). In recent years, the mean daily temperatures have risen in Hanoi, likely resulting from climate change and urbanization [41].

Meteorological observations of the daily 24-hour ambient temperature were collected from several monitoring stations in Hanoi. The different monitoring stations were summed to estimate an average of a city mean value of daily maximum temperature. We applied the daily values of ambient maximum temperature to examine its association with the rates of mental diseases over the study period of 2008 to 2012.

### Statistical analysis

The daily frequencies of cases with psychiatric problems were aggregated per disease category and per groups of age, sex and location on a daily basis over the study period. We established zero-inflated negative binomial time series regression models of the aggregated counts of daily admissions as an outcome variable and indicator variables for: extreme heat of one, three or seven consecutive days, the day of the week, season, and the time trend in the study period as explanatory variables.

A set of definitions for heatwaves is presented in the literature covering both absolute and relative values, using varied combinations of indicators. However, most of the definitions are based on the condition of meteorological aspects, the relative occurrence of the event, and fewer of them are based on physiological thresholds for thermal stress [42, 43]. In particular, none of them have been developed to capture mental stress physiological reactions and mechanisms. During five years (2008–2012), the average maximum temperature in Hanoi was about 32°C during the hot months (May to September), with a standard deviation of ± 3°C. In this study, we defined heatwaves using consecutive days of a temperature threshold beyond normal skin temperature (above 34°C). This definition corresponded to the 90^th^ percentile of daily maximum temperature. In China, Li Bai et al. found that maximum temperature was a better predictor of heat-related illnesses in summers compared with a heat index [44]. Moreover, we found the physiological definition of ambient temperature exceeding skin temperature appealing and estimated the 90^th^ percentile to the same threshold; other recent publications have defined heatwaves using consecutive days of varying temperature thresholds. In Canada, Wang et al. found a strong relation between emergency room visits for mental disorders and a mean daily temperature of 28°C, covering both the cold and warm seasons [25]. In addition, Hansen et al. defined heatwaves in Adelaide, Australia as three or more consecutive days of a maximum temperature reaching or exceeding 35°C [7]. Some researchers have argued that it is important to establish an appropriate definition of heatwave locally in relation to its health impacts. However, it appears desirable to have a dynamic and flexible heatwave definition when population characteristics, epidemiological profiles of populations and weather conditions may change over time and modify health impact estimates [42]. Thus, in the present study, a definition of heatwaves similar to that of other studies was used to estimate the effect of heatwaves in relation to hospital admissions for mental diseases.

As a sensitivity analysis we also investigated relative risks related to definitions of the 95^th^ and 99^th^ percentiles of daily maximum temperature. Seasons were defined as spring (March to May), summer (June to August), autumn (September to November) and winter (December to February). We additionally assessed the sensitivity of relative risks estimated when adjusting for the season and time trend as one natural cubic spline function of time with 16 degrees of freedom. Furthermore, hospital admissions for mental disorders were analysed collectively and with stratification by the seven disorder groups, age groups (age: 0–17; 18–40; 41–60; and ≥ 61), sex, and location.

Estimates of relative risks (RRs) were derived and complimented by computation of 95% confidence intervals (CIs). Statistical analyses were conducted using the statistical software package Stata v13.

### Ethics Statement

Electronic health data sets, including the code, age, sex, date, and treatment of admissions for mental disorders, both children and adults, were anonymized and de-identified prior to analysis. All procedures were approved by the Hanoi Medical University and the Mental Hospital Ethics Board in Vietnam.

## Results

### Weather variation

**Fig 1:**
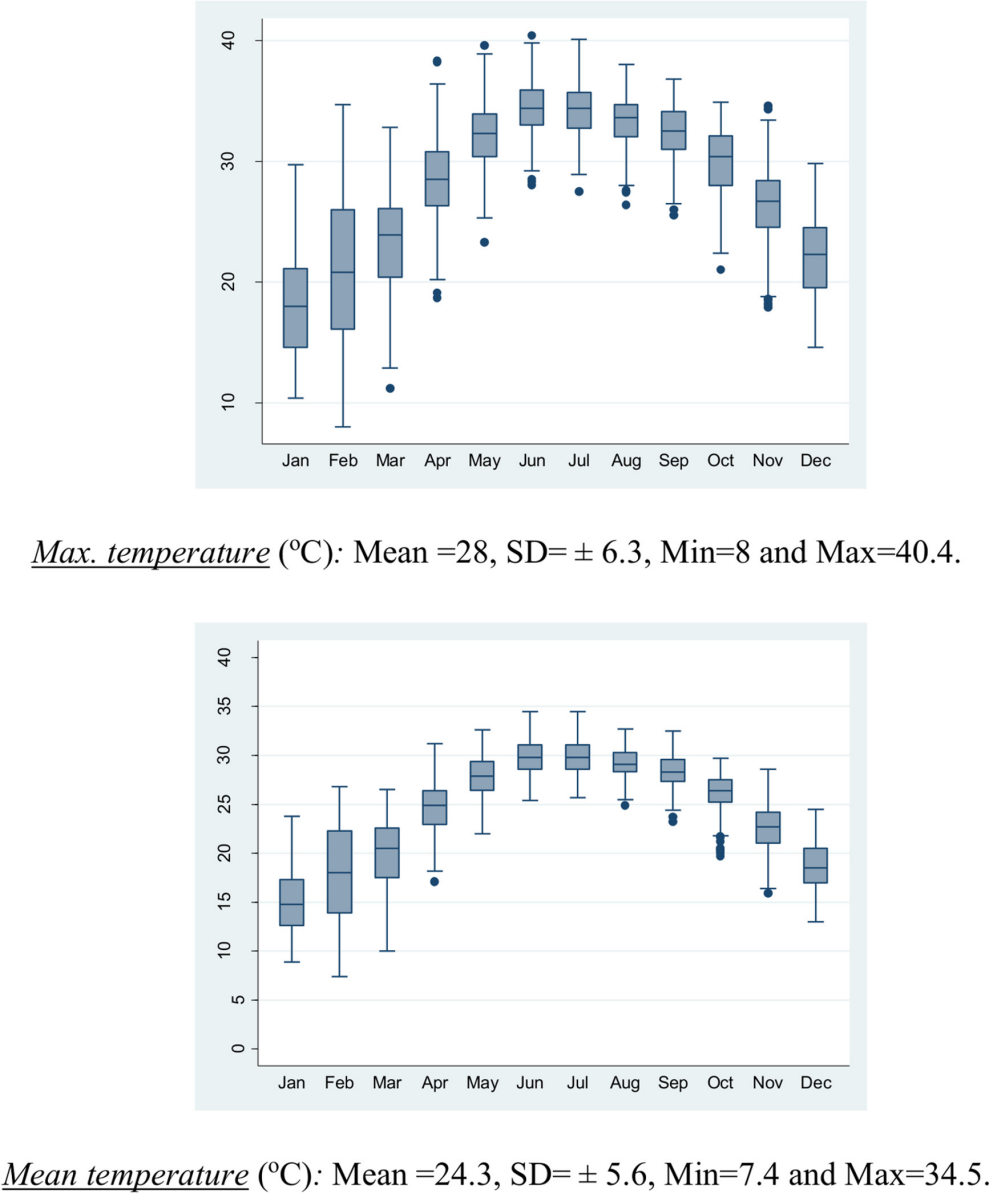
The average of monthly maximum, mean, and minimum temperatures as well as mean humidity during five years. The temperature and mean humidity variances followed the month trend over five year (2008–2012). The hottest weather was during summer season (Jun–Aug).

**Fig 2:**
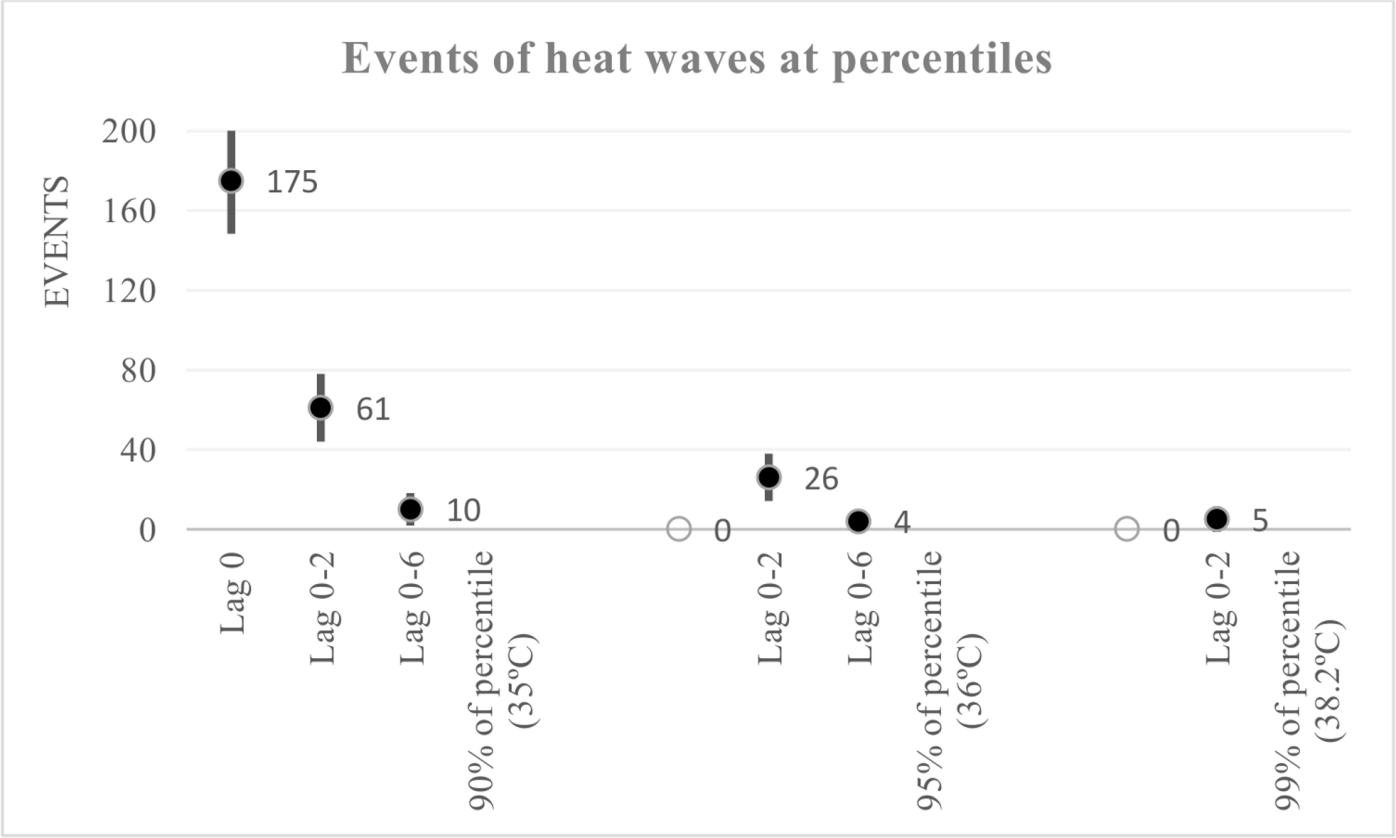
Events of heatwaves at percentiles during five years 2008 to 2012 in Hanoi. There were 175 single day, 61 events at ≥ three consecutive days, and 10 events at ≥ seven consecutive days with maximum temperature exceeding 35°C (90^th^ percentile).

The annual averages of the daily minimum and mean temperatures during the period 2008–2012 were about 21.8 ± 5.2°C and 24.3 ± 5.6°C (standard deviations), respectively. In addition, the mean humidity in Hanoi during the 5 years was around 78% ± 10% (41%– 98%). The annual average of the daily maximum temperature was around 28°C with a maximum of 40.4°C and a minimum of 8°C. The monthly mean temperature in the winter season from December to February was the lowest, with a daily maximum temperature estimated on average at between 18 and 21°C. Contrastingly, the maximum temperature in the warm season, from May to September, was the greatest and averaged to 32°C with a standard deviation of ± 3°C (Fig 1). In this study, over 5 years, 175 single days were observed with a maximum temperature exceeding 35°C (90^th^ percentile). Of those, 61 events included at least three consecutive days of such conditions, and 10 events included at least seven consecutive days of such temperatures (Fig 2).

### Hospital admissions for mental disorders

Throughout the period of the study there were 21,443 hospital admissions at the mental hospital. Of those, 18,075 (84.3%) were inpatients and 3,368 (15.7%) were outpatients. A total of 16,327 (76.1%) were male patient and 5,116 (23.9%) were female patients. Urban residents accumulated 12,547 visits (58.5%) and rural residents accumulated 8,874 visits (41.4%). In the age groups: 0–17; 18–40; 41–60; and ages above 60 years, 655 (3.0%), 10,512 (49.0%); 8,681 (40.5%); and 1,595 (7.5%) hospital admissions, respectively, were observed. Moreover, 96% and 4% of patients with mental disorders were admitted in the hospital daily between 6 a.m and 6 p.m and between 7 p.m and 5 a.m, respectively. From 6 a.m to 6 p.m, temperatures were usually quite high, especially in the spring and summer seasons (Table 1).

**Table 1:** Descriptive statistics of the study population of 21,443 patients with a median age of 40.8 ± 13.6 years admitted for mental disorders at the Hanoi Mental Hospital from 2008 to 2012.

	*Frequency*	*Per cent (%)*
*Ages (years)*		
< 18	655	3.0
*18–40*	*10512*	*49*
41–60	8681	40.5
> 60	1595	7.5
*Sex*		
Female	5116	23.9
*Male*	*16*,*327*	*76*.*1*
*Location*		
*Urban*	*12*,*547*	*58*.*5*
Rural	8874	41.4
Unknown	22	0.1
*Patients*		
*Inpatients*	*18*,*075*	*84*.*3*
Outpatients	3368	15.7
Admitted in hospital from 6 a.m. to 6 p.m.		
Patients	20,590	96%

**Fig 3:**
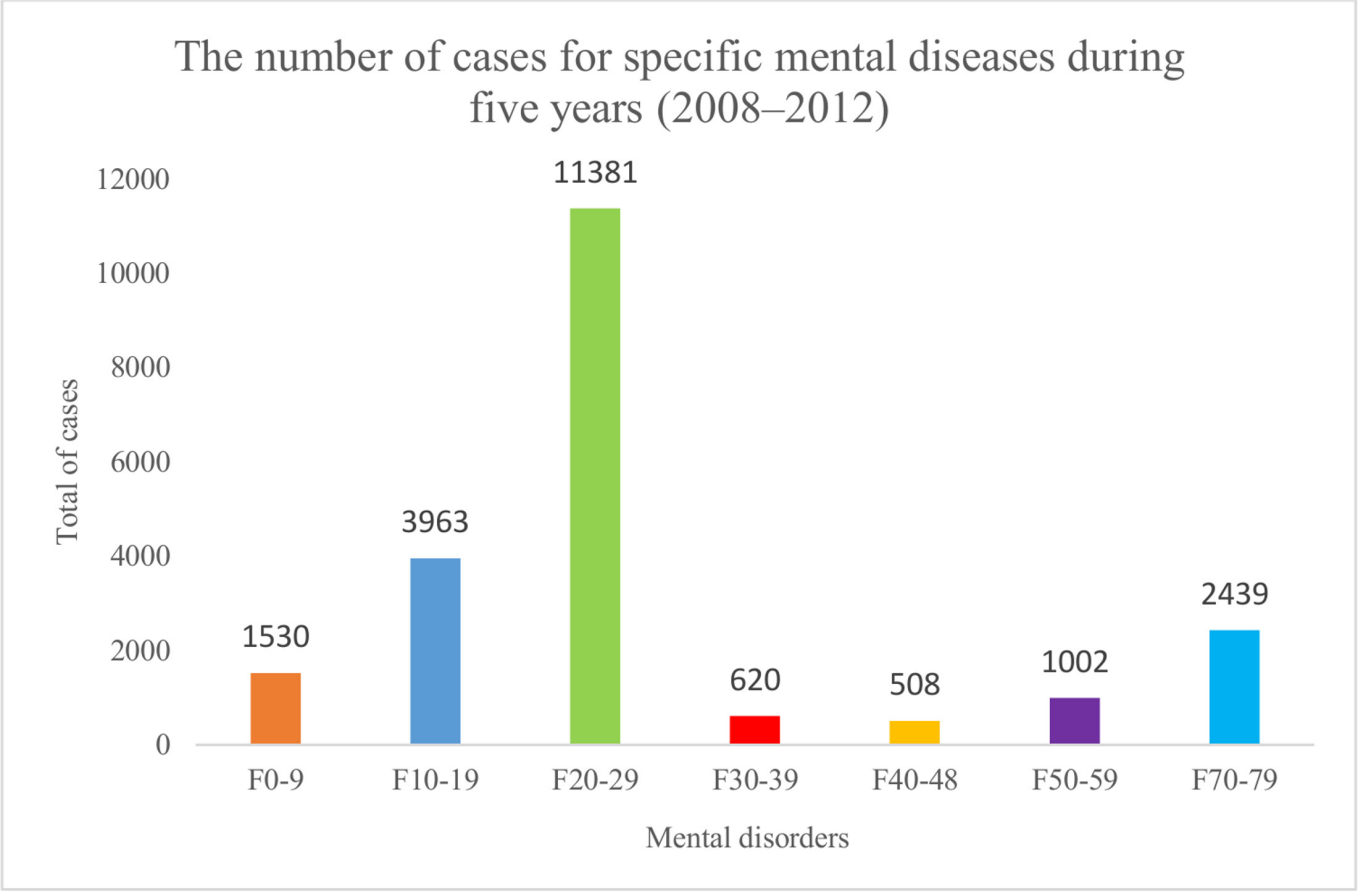
The number of cases for specific mental diseases during five years. The group of F20-29 accounted for the highest hospitalizations (11381), and the group of F40-48 accounted for the lowest admissions (508).

The group of schizophrenia, schizotypal, and delusional disorders (F20-29) accounted for the highest proportion (53.1%) of hospitalizations, and the group of neurotic, stress-related, and somatoform disorders (F40-48) (2.4%) accounted for the lowest (Fig 3).

### The association between heatwaves and hospital admissions for mental disorders

#### All mental disorders

**Fig 4:**
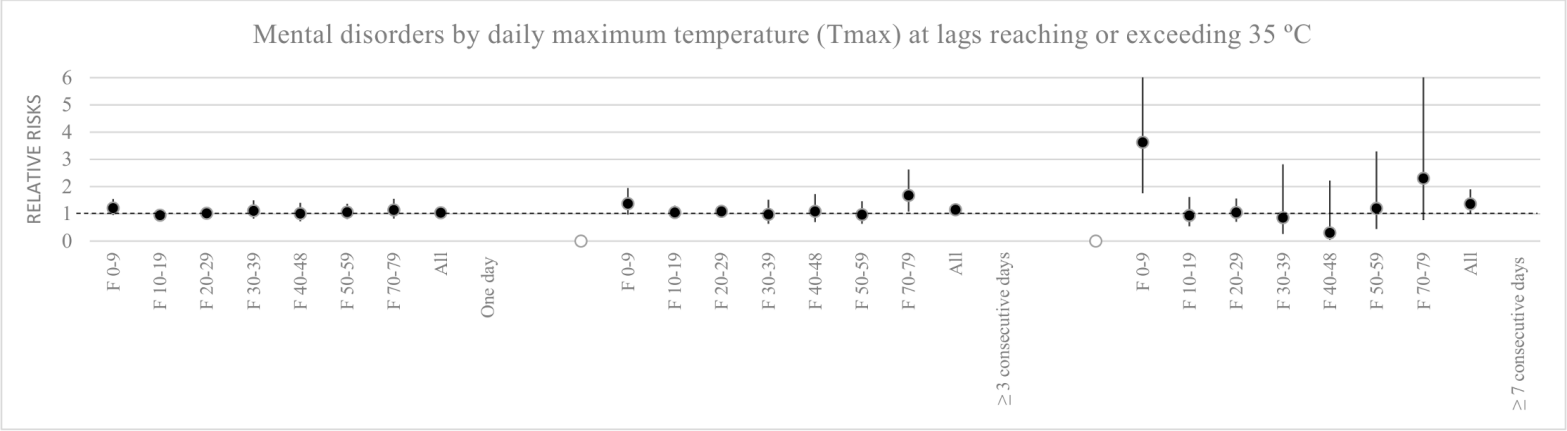
Mental disorders by daily maximum temperature at lags reaching or exceeding 35°C. The number of cases for mental disorders increased with the length of the heatwave with relative risk estimating to 1.04 (0.95–1.13), 1.15 (1.005–1.31) and 1.36 (1–1.90) for one day, at least three days, or seven days in a row.

**Fig 5:**
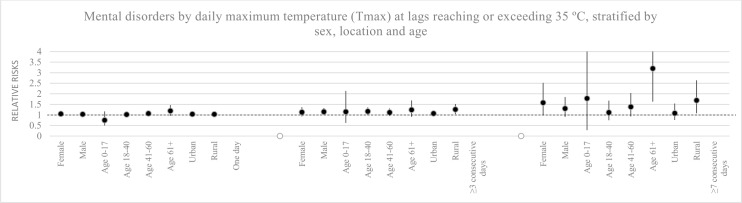
Mental disorders by daily maximum temperature at lags reaching or exceeding 35°C, stratified by sex, locations and age groups. During heatwaves at ≥ 3 consecutive days, admissions for mental disorders among rural populations and among men estimated a relative risk of 1.26 (1.04–1.52) and 1.15 (1–1.33). During heatwaves at ≥ 7 consecutive days, cases for the age group above 60 years and residents in rural areas increased with RRs estimating to 3.2 (1.63–6.29) and 1.69 (1.08–2.64).

We estimated an association between temperatures and admissions for mental disorders with relative risks of 1.02 (1.007–1.02), 1.02 (1.007–1.03), and 1.02 (1.004–1.03) when maximum, mean, as well as minimum temperatures increased by one degree Celsius (Table 2). When the temperature exceeded 35°C of maximum temperature for one day, at least three days, or seven days in a row, the number of cases for mental disorders increased with the length of the heatwave with RR estimating to 1.04 (0.95–1.13), 1.15 (1.005–1.31) and 1.36 (1–1.90), respectively (Fig 4). During heatwaves of at least three consecutive days, admissions for mental disorders among rural populations and among men estimated a relative risk of 1.26 (1.04–1.52) and 1.15 (1–1.33), respectively. During heatwaves of at least seven consecutive days, the age group above 60 years and residents in rural areas showed the strongest associations to heatwaves, with RRs estimating to 3.2 (1.63–6.29) and 1.69 (1.08–2.64), respectively (Fig 5).

**Table 2:** The relationship between temperatures and daily hospital admissions for mental disorders during five years (2008–2012).

*Admissions*	*Relative risks*	*Confidence interval (95%)*
Max. temperature	1.02	1.007–1.02
Mean temperature	1.02	1.007–1.03
Min. temperature	1.02	1.004–1.03

#### Specific mental disorder

We found hospital visits for mental diseases being differently associated with heatwaves depending on the type of mental disorder diagnosis. The group of mental retardation (F70-79) had the highest relative risk associated with heatwaves of three consecutive days amounting to 1.68 (1.08–2.62). There was also a strong relationship between heatwaves of at least seven consecutive days and admissions in organic mental disorders including symptomatic illnesses (F0-9) with the relative risk amounting to 3.62 (1.7–7.42) (Table 3).

**Table 3:** Admissions for F0-9 and F70-79 by daily maximum temperature at lags reaching or exceeding 35°C, stratified by sex, location, and age groups.

	*Mental disorders for F0-9 and F70-79 by daily maximum temperature (T-max) at lags*, *reaching or exceeding 35°C*, *stratified by sex*, *location and age*		
	*Lag 0*	*Lag ≥3 consecutive days*	*Lag ≥7 consecutive days*
*Organic*, *including symptomatic*, *mental disorders (F0–9)*: *RRs (95% CI)*			
F 0–3	0.93 (0.64–1.36)	1.01 (0.6–1.7)	1.85 (0.6–6)
F 4–6	1.31 (0.94–1.82)	1.52* (1–1.004)	4.76* (1.74–13.18)
F 7–9	1.03 (0.6–1.84)	0.96 (0.42–2.19)	0.8 (0.1–7.4)
F 0–9	1.21 (0.95–1.54)	1.37 (0.97–1.95)	3.62* (1.76–7.42)
Female	0.88 (0.63–1.24)	1.23 (0.8–1.9)	2.11 (0.9–5.06)
Male	1.37 (1–1.9)	1.4 (0.86–2.27)	6.3* (2.1–19.05)
Age 0–40	0.88 (0.61–1.27)	1 (0.6–1.71)	0.89 (0.18–4.47)
Age 41–60	1.32 (0.85–2.04)	1.49 (0.84–2.66)	4.05* (1.51–10.88)
Age 61+	1.6* (1.1–2.34)	1.55 (0.92–2.62)	5.82* (2.09–16.25)
Urban	0.94 (0.69–1.29)	0.82 (0.5–1.36)	0.93 (0.24–3.51)
Rural	1.41* (1–2.01)	2* (1.23–3.27)	7.82* (2.66–22.96)
*Mental retardation (F70–79)*: *RRs (95% CI)*			
F 70–71	1.18 (0.86–1.62)	1.75* (1.11–2.76)	2.69 (0.9–8.14)
F 72–73	0.9 (0.5–1.63)	1.33 (0.58–3.05)	0.3 (0.02–5.36)
F 70–79	1.14 (0.83–1.55)	1.68* (1.08–2.62)	2.3 (0.8–6.88)
Female	1.27 (0.86–1.88)	1.65 (0.95–2.85)	3.53* (1–12.47)
Male	1.07 (0.75–1.52)	1.65* (1.001–2.73)	1.99 (0.6–7.08)
Age 0–17	0.95 (0.5–1.84)	1.36 (0.56–3.32)	1.82 (0.2–16.86)
Age 18–40	1.14 (0.8–1.62)	1.84* (1.12–3.02)	2.29 (0.66–7.89)
Age 41+	1.38 (0.88–2.16)	1.63 (0.86–3.07)	3.2 (0.72–14.18)
Urban	1.16 (0.8–1.69)	1.83* (1.11–3.04)	2.06 (0.7–6.97)
Rural	1.14 (0.8–1.67)	1.63 (0.94–2.85)	2.61 (0.65–10.47)

(* p < 0.05)

Although there were increased trends in the rates of admissions for three and seven consecutive days for six of the seven specific mental disease groups, we found no statistically significant associations in the other groups. The number of cases for schizophrenia, the largest sub-category of mental disorders, indicated an increased RR of 1.09 (0.94–1.27) for heatwaves of at least three consecutive days (Fig 4).

#### Organic mental disorders including symptomatic illnesses

**Fig 6:**
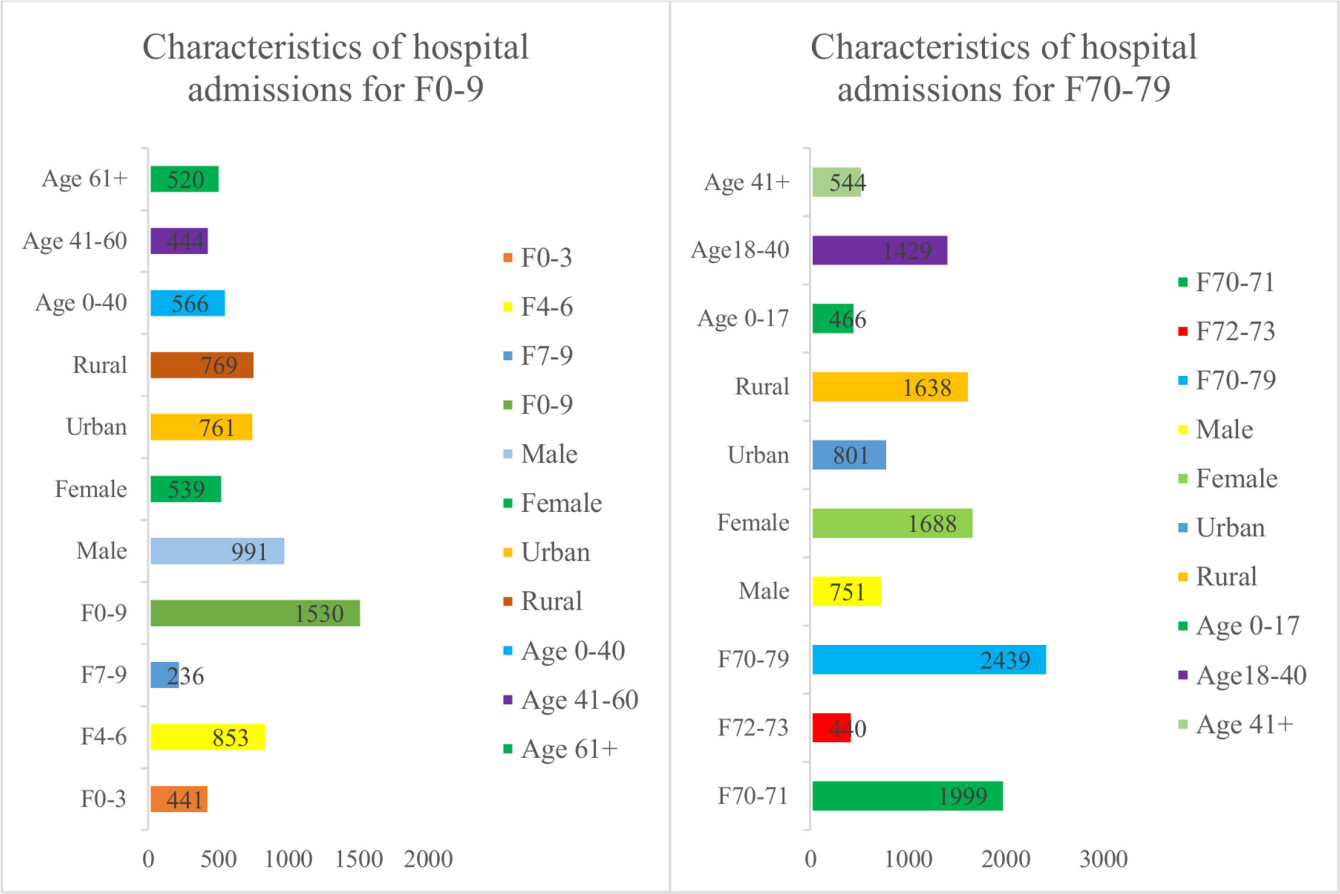
Descriptive statistics of the admissions for F0-9 and F70-79 at the Hanoi Mental Hospital from 2008 to 2012. Admissions for F0-9 among male patients were higher than others; while cases for F70-79 among female patients were greater than others.

The group of organic mental disorders including symptomatic illnesses (F0-9) were divided into F0-3 (dementia), F4-6 (organic amnesic syndrome, delirium; other mental disorders due to brain damage) and F7-9 (personality and behavioural disorders due to brain disease, damage and dysfunctions) and stratified by sex, age and location. There were 1530 admissions for F0-9 during the study period of 2008–2012, in which F0-3, F4-6, F7-9 accounted for 441 (28.8%), 853 (55.8%), and 236 (15.4%) admissions, respectively. There were 991 males (64.8%), 539 females (35.2%), 761 residents in urban areas (49.7%), 769 residents in rural areas (50.3%), 566 subjects were aged below 40 years (37.0%), 444 were aged 41–60 years (29.0%), and 520 were aged over 60 years (Fig 6). The hospital visits for mental disorders in this group showed a strong relationship to heatwaves. In this study, the results showed that for extreme heat lasting at least one day, three days, and seven consecutive days, the highest relative risks were associated with heatwaves of at least seven consecutive days. In particular, in heatwaves of at least seven days, high relative risks were observed in the groups F4-6; males; aged 41–60 years; aged over 60 years; and residents in rural areas, amounting to 4.76 (1.74–13.18), 6.3 (2.1–19.5), 4.05 (1.51–10.88), 5.82 (2.09–16.25) and 7.82 (2.66–22.96), respectively. Overall, the results indicated that admissions followed a pattern of increased relative risk when the duration of the heatwave was from one day to seven days, and that ages over 60 years and residents in rural areas accounted for the highest relative risk increases (Table 3).

#### Mental retardation

The group of mental retardation patients (F70-79) was divided into sub-disease groups of F70-71 (mild and moderate intellectual disabilities) and F72-73 (severe and profound intellectual disabilities), and were stratified by sex, age and location. There was a total of 2,439 admissions for F70-79, of which F70-71 and F72-73 numbered 1,999 (82.0%) and 440 (18.0%) cases, respectively. Among those 1,688 were males (69.2%), and 751 were females (30.8%), 801 were residents in urban areas (32.8%), and 1,638 were residents in rural areas (67.2%), 466 were subjects aged 0–17 years (19.1%), 1,429 were aged 18–40 years (58.6%), and 544 were aged over 40 years (22.3%), (Fig 6). The results showed that hospital visits for F70-79 had significant correlations to heatwaves, especially heatwaves of at least three consecutive days of extreme temperatures. There were high relative risks among males, people aged 18–40 years, and residents in urban areas, with RRs estimating to 1.65 (1.001–2.73), 1.84 (1.12–3.02), and 1.83 (1.11–3.04), respectively. When comparing the relative risk associated with the same groups at only one day of extreme temperature with extreme temperature of at least three or seven consecutive days, there were increased trends with the longer heat events, particularly among F70-71, with relative risks 1.18 (0.86–1.62), 1.75 (1.11–2.76) and 2.69 (0.9–8.14), respectively (Table 3).

### Hospital admissions for mental disorders in relation to heatwaves at 95^th^ and 99^th^ percentiles of maximum temperature

**Fig 7:**
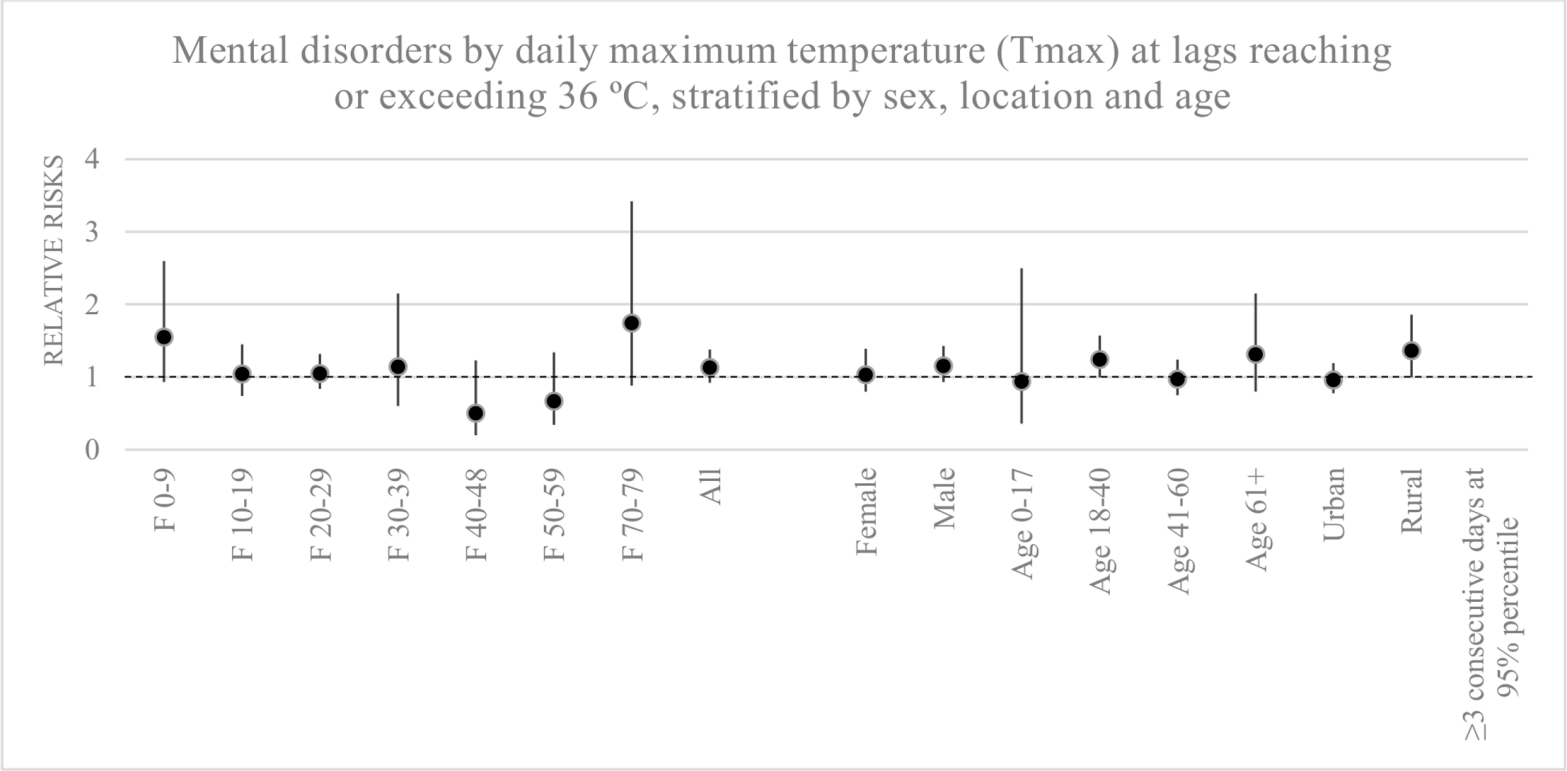
Mental disorders by daily maximum temperature at lags reaching or exceeding 36°C (at 95^th^ percentile of maximum temperature) stratified by sex, locations and age groups. Hospital visits in the groups F0-9, F70-79, males, and residents in the rural area were affected by heatwaves more than others.

There were no significant relationships between admissions for mental disorders and heatwaves of at least seven consecutive days of the 95^th^ and at least three consecutive days of the 99^th^ percentiles of maximum temperature. However, the number of cases for mental disorders increased at least three days of the 95^th^ percentile, with the relative risk of 1.13 (0.92–1.38) (Table 4). In particular, in the groups F0-9; F70-79; males; aged 18–40 years; aged over 60, and residents in rural areas, risks were elevated with RR of 1.55 (0.93–2.6), 1.74 (0.9–3.42), 1.15 (0.93–1.43), 1.24 (1–1.57), 1.31 (0.8–2.15), and 1.36 (1–1.86), respectively (Fig 7).

**Table 4:** Admissions for mental disorders by daily maximum temperature at lags at 95^th^ (36°C) and 99^th^ (38.2°C) percentiles of maximum temperature.

Percentiles (%)	Temperature (°C)	Admissions for mental disorders Relative risks (95% CI)	
95^th^	36°C	Lag ≥ 3 consecutive days	1.13 (0.92–1.38)
		Lag ≥ 7 consecutive days	0.91 (0.5–1.74)
99^th^	38.2°C	Lag ≥ 3 consecutive days	0.84 (0.53–1.35)
		Lag ≥ 7 consecutive days	-

Furthermore, adjusting for seasonality and time trends using a cubic spline function with 16 degrees of freedom instead of month and year indicators did affect the estimates, but patterns and relative risks remained (Table 5).

**Table 5:** The difference of estimates between: adjusting seasonality, time trends using a cubic spline function and only controlling month, year indicators.

Patterns	Results	
Admissions, heatwaves, years, months, day of week	Lag 0	1.05
		(0.96–1.15)
	Lag ≥3 consecutive days	(1.01–1.33)1.16
		
	Lag ≥7 consecutive days	1.42
		(1.02–1.99)
Admissions, heatwaves, time trends, day of week, daily mean humidity	Lag 0	1.04
		(0.95–1.13)
	Lag ≥3 consecutive days	1.15
		(1.005–1.31)
	Lag ≥7 consecutive days	1.36
		(1–1.90)

## Discussion

We found associations between heat exposure and hospitalizations for mental illness in Northern Vietnam with relative risks increasing with the length of the heatwaves. Moreover, the groups of organic mental illness as well as those with mental retardation had the highest increase during these events. We saw impacts among both males and females, in all age groups. It appeared that rural residents in particular suffered more from longer heat-heatwaves. These findings from a population and society undergoing rapid development and change in terms of epidemiological and demographic transition call for public health interventions to protect populations from mental illnesses.

A relationship between mean, minimum, as well as maximum temperatures and mental disorders was found with the same relative risks of 1.02, but with the highest precision for associations with maximum temperature. The choice and definition of the heatwave threshold in the study was found to be important, with significant associations with the 90^th^ percentile, but not with the 95^th^ and 99^th^ percentile definitions of maximum temperatures. However, the number of events, especially for consecutive heatwave periods, for the 95^th^ and 99^th^ definition were almost impossible to study due to the limited number of events and correspondingly low power.

The fact that longer heatwaves resulted in a higher relative risk deserves to be mentioned and explained. In this study, over five years, 175 single days was observed with the maximum temperature exceeding 35°C. Of those events, 61 included at least three consecutive days with such conditions, and ten events of these included at least seven consecutive days with such temperatures. These days amounted to increases of admissions by 4%, 15% and 36% compared with non-heatwave conditions, respectively. This suggests that the duration of heatwaves magnifies the impact on mental health. This has also been previously found for other disease causes and mortalities [45]. With regard to mortality, in Sweden persons with a pre-existing hospital admission in a mental disorder were found to have the highest relative risk of death as the duration of heatwaves lengthened [46]. We found a similar pattern, with mental diseases indicating elevated relative risks, particularly in the F0-9 group, which increased sharply with an RR of 3.62 (1.76–7.42) at ≥ seven consecutive days of heatwave. The reason persistent exposure causes more harm than temperature indicators themselves has been discussed in earlier studies. Studies of mortality have previously suggested additional impacts during heatwave are not explained by the temperature itself alone, but due to the duration of the heatwave [47]. Rocklöv et al. explained that additional heatwave effects could technically occur by the exclusion of temperature lag interactions in time series studies [48]. However, the occurrence of such an effect has also been debated, and some studies do suggest that the temperature–disease relationship and lag effects can be made complex enough to capture most of the ‘added’ heatwave effect [49]. Overall, our study indicates the length of a heatwave to be of particular importance, at least for the impacts on hospital admissions for mental disorders. This could be a dose-response effect of persistent heat exposure causing cumulative stress leading to mental problems/exacerbation.

Published literature has shown populations with mental illness as being a vulnerable and heat-susceptible group in addition to children, the elderly and residents in rural areas [4, 6, 7, 15, 18]. However, publications on heat-related mental disorders and exacerbations have previously been based on populations in developed nations [7,12, 17]. A study on heatwaves and mental health in a temperate Australian city showed high rates of mental illness related to heatwaves of at least three consecutive days of the threshold of 35°C maximum temperature, and estimated an increase by 7.3% (RR = 1.073, 95% CI = 1.017–1.132), among which disorders in the group F0-9 accounted for the majority of cases (RR = 1.213, 95% CI = 1.091–1.349) [7]. The results have many similarities with our findings, and may show a global pattern of mental health susceptibility to heatwaves. In addition, a study conducted in France on psychotropic drug use and the risk of heat-related hospitalizations indicated associations between using anticholinergic drugs, antipsychotics, and anxiolytic drugs during heatwaves, with ORs of 6.0, 4.6, and 2.4, respectively [45]. Heat stress influenced mood disorder, psychological distress and mental health problems, and caused declining human psychological performance [5,16, 21]. The findings of a study using self-reported health and well-being in Thailand suggested that climate-related heat stress existed as well as daily activities such as sleep, housework, and exercise [50]. The effect of daily weather variables on psychosis admissions to mental hospitals, according to the Irish Health Research Board indicated that there existed a weak relationship between temperature and psychosis admissions [21]. However, the temperatures in Ireland seldom reach 35°C, which was used to define heat events in this study. There were acute impacts of extreme temperature exposure on emergency room admissions for mental and behaviour disorders in Toronto, Canada with a strong association at 28°C. The strongest association was seen within a period of 0–4 days of exposure to hot temperatures. There was an increased trend in mental disorders with a relative risk of 1.29 (95% CI = 1.09–1.53) over a cumulative period of seven days after exposure to an ambient temperature at the 99^th^ percentile [25].

In this study, the relationships between heatwaves and disease in the sub-category F0-09 are related to several transitions of social and demographic determinants in Vietnam. First of all, populations are growing older and groups above 60 years of age in Vietnam show a higher risk of dementia [51]. The predisposing factors for the development of delirium (F4-6) are pre-existing brain damage, alcohol dependence, and others. The number of patients with damaged brains due to traffic accidents has been increasing, especially among males [52]. In addition, the prevalence of alcohol use disorders and alcohol dependence in men over 15 years of age has been estimated at 8.7% and 5.9%, respectively [53]. Moreover, being male is an independent risk factor for delirium (according to DSM IV TR) [54]. The result of this study showed that admission for delirium is particularly elevated during longer heatwave episodes.

Moreover, the number of hospital admissions for sub-category F70-79 had high risks during the periods of heatwaves. According to published literature, patients with mental retardation usually visit mental hospitals due to psychological problems in which the most frequent reasons included aggression, appearance or worsening of psychotic symptoms, worsening or changing behaviours [55]. In addition, there was an estimate of the prevalence of comorbidity between psychiatric disorders and mental retardation in community and clinical populations, with a range from 14.3 to 67.3 per cent including common cases of schizophrenia, mood disturbances and anxiety [56]. In Vietnam, around one million people with mental retardation are taken care of by the mental health services [57]. Thus, the risk of admissions for mental retardation may be high during hot weather. This may sound an alarm for mental health managers to set up strategies to prevent mental health problems, especially during the periods of high temperatures.

Although six of seven specific mental diseases (F0-9, F10-19, F20-29, F30-39, F40-48, and F50-59) had insignificant correlations to heatwaves, there were increased trends in the risk of admissions for three and seven consecutive days of heatwaves. Schizophrenia, in particular had a high risk, with RR of 1.09 (0.94–1.27) for heatwave of at least three consecutive days’ duration. The results of studies in Israel (R = 0.35, p < 0.001), and Canada (RR = 1.1, 95% CI = 1.03–1.17) showed that persistent high environmental temperature may play a contributing factor in psychotic exacerbation in patients with schizophrenia [23, 25].

In the present study, age and gender as risk factors for heat vulnerability have been widely examined. The elderly may have a low heat tolerance, depending on age-related conditions in both their physiological capability to regulate temperature and general health, which may induce increased risk of heat-associated illness [58–60]. The number of admissions for mental disorders (F00-79) in the older group of over 60 years of age had an increased trend with longer exposure to heat extremes. Considering a specific mental disease like F0-9, the association between age and heat vulnerability had a high risk in the elderly over 60 years at respective strata. Meanwhile, there was an increase in hospital visits for F70-79 among the age groups of 18–40 years and over 40 years at ≥ three consecutive days. The relationship between gender and heat vulnerability varies across situations and nations. Literature has shown that women are at higher relative risk of death in most European countries, but evidence in the United States indicates that men are at higher risk of death during heatwaves [54]. In our study, there was a significant difference in the gender distribution between the two sexes, in which the number of cases for males was greater than for females according to the frequency of events. Admissions for mental disorders among men were threefold compared to those among women. According to the literature of psychiatric disorders, male mental patients are more common than female, except for patients with depressive disorders [54]. A personal interview survey on common mental disorders with questionnaires in the north of Vietnam also showed a higher rate of psychiatric disorders amongst male patients than amongst female patients [61]. Greater proportions of alcohol consumption and brain damage due to traffic accidents among men in Vietnam may explain in part the increased risk in mental disorders for male patients [52, 53]. In addition, males were more at risk in heatwaves than females. In terms of gender and heatwaves in relation to specific mental diseases, the admissions among males had higher estimated risks compared to the females. Moreover, men spend more time outside in occupational activities compared to women [62]. This may explain the high relative risks associated with high temperature.

Furthermore, living environment and conditions may affect health status in general, as well as mental disorders in particular. In this study, admissions among residents in rural areas had significant differences from those in urban areas in heatwave events of at least three and seven days. Hanoi is not a very large city, so different temperatures are not common between districts and suburbs. Differences between urban and rural areas may be related to occupational activities. Vietnam is an agricultural nation, with famers in suburbs and rural areas. This may explain that residents in rural areas working outdoors are exposed to high temperatures for longer than others. With regards to specific mental disorders of the F0-9 group, there was an increased trend in cases with long heatwaves among residents in rural areas, with RRs of 1.41 (1–2.01), 2 (1.23–3.27), and 7.82 (2.66–22.96) at strata one day, ≥ three, and ≥ seven consecutive days, respectively. While the number of cases among populations in urban areas had a decreasing trend with heatwaves, conversely, hospital visits for F70-79 among persons in urban areas were at risk of experiencing more heatwaves than those in rural areas, especially at ≥ three consecutive days. Moreover, there was an increased trend in admissions for mental disorders with RR of 1.36 (1–1.86) among residents in rural areas during heatwaves lasting at least three consecutive days when maximum temperatures reached 36°C corresponding to the 95^th^ percentile. Thus, patients with mental health problems in the rural area were more sensitive to the hot weather than those in the urban area. In this study, because the mean maximum temperature was estimated as the same value for both urban and rural areas, this is a limitation of our study. It is necessary to conduct further research on mental health problems and environmental conditions to clearly understand the differences in admissions for mental disorders between rural and urban areas in Vietnam.

In the present study, although we mainly interpreted heatwaves at a threshold beyond 35°C corresponding to the 90^th^ percentile of mean maximum temperature, we also analysed the relationship between admissions for mental disorders and heatwaves at ≥ three consecutive days of the 95^th^ percentile of maximum temperature. The study results also similarly indicated hospital visits in the groups F0-9, F70-79, males, and residents in the rural area were affected by heatwaves more than others. Furthermore, the use of a cubic spline function of time has been favoured to adjust for seasonality and time trends aimed at controlling additional confounding effects [63]. However, since using data from a mental hospital may not capture all mental disorder admissions, further studies need to be carried out to collect fully cases of psychiatric and behavioural disorders.

Considering previous studies on the relationship between high temperature exposure and physiological mechanisms, several experimental studies have indicated that an increase in core body temperature following heat stress is related to a rise in brain temperature, because there is an increase in heat production in the brain and/or changed neuronal metabolic activity responsible for impaired neuronal activity and brain dysfunction [31]. Kiyatkin et al. demonstrated that brain temperature varies in distinct regions from 0.5°C to 1.5°C as well as being significantly influenced by external environmental conditions such as alteration to ambient temperature [64]. One of the most crucial brain functions impacted by hyperthermia is a change in the blood–brain barrier permeability. A selective disruption of the blood–brain barrier to proteins in the areas displaying brain oedema formation and breakdown of blood–brain barrier is instrumental in heat-related brain dysfunction [31]. Neurotransmitters, including serotonin, dopamine, catecholamine, GABA (Gamma Aminobutyric Acid) and others, are related to mental problems [54]. J.V. Christman and colleague conducted a study on the role of norepinephrine in the anterior hypothalamus where the results showed a significant increase in norepinephrine in the preoptic area and DOPEG (dihyroxyphenylglycol). The data indicated that exercise in the heat (at ≥ 35°C) increased norepinephrine-induced peripheral heat-dissipating capacity as well as inducing a rise of catecholamine storage in the preoptic area [65]. Recent studies proved that a combined inhibition of norepinephrine/dopamine reuptake improved exercise performance in high ambient temperature (warm environment ≥ 30°C) as well as increasing core temperature in heat [34, 35]. Furthermore, the environmental thermal condition contributes to the regulation of the internal body temperature. Researchers demonstrated that small changes in ambient temperature (2°C) can lead to changes in the thermal response to 3,4-methyl dioxymethamphetamine in which at 30°C there was a clear hyperthermic effect, co-occurring with a decrease in serotonin concentration [35]. Findings related to a physiological mechanism between high temperature and psychiatric disorders as well as brain temperature may contribute to evidence explaining the hypothesis in this study that hospital admissions for mental disorders may be triggered or exacerbated by heatwaves. However, further research on biological, psychological and physiological areas is required to determine the exact mechanism of the relationship between mental disorders and environmental conditions such as extreme heat or heatwaves.

The results call for more attention, and specific action and adaptations measures to be taken, to protect future vulnerable and susceptible populations under epidemiological transition and environmental changes.

## Conclusions

This study demonstrated relationships between admissions for mental disorders and heatwaves, especially at a threshold temperature beyond 35°C. The rates of admissions were found to increase substantially with the length of the heatwaves. There were large differences between gender, urban and rural residents, and age groups. Men, residents in rural regions, and elderly populations over 60 years of age appeared to face the largest relative risks of being admitted for mental disorders in periods of heatwaves. In addition, admissions for organic mental illness and mental retardation showed the strongest significant associations with heatwave events. The research results pave the way for showing the need for further studies and public health measures to protect populations already at risk from the consequences of climate and environmental changes.

### Limitations of the study

The study used hospital registry data from one psychiatric hospital in northern Vietnam. This may not capture fully all mental disorder patients in Hanoi, especially outpatients. In addition, it is not possible to generalize the results nationwide because Vietnam has different areas with distinctly specific weather patterns. It is essential to carry out more studies in the future using sufficient information and to set up a model including environment, personal trait, and socio-economics, together with admissions for mental health disorders.

## Supporting Information

S1 Dataset Stata format.dta. (DTA)

S1 Table Dataset (Excel format).xls. (XLS)
